# Dating the Origin of Language Using Phonemic Diversity

**DOI:** 10.1371/journal.pone.0035289

**Published:** 2012-04-27

**Authors:** Charles Perreault, Sarah Mathew

**Affiliations:** 1 Santa Fe Institute, Santa Fe, New Mexico, United States of America; 2 Centre for the Study of Cultural Evolution, Stockholm University, Stockholm, Sweden; University of Oxford, United Kingdom

## Abstract

Language is a key adaptation of our species, yet we do not know when it evolved. Here, we use data on language phonemic diversity to estimate a minimum date for the origin of language. We take advantage of the fact that phonemic diversity evolves slowly and use it as a clock to calculate how long the oldest African languages would have to have been around in order to accumulate the number of phonemes they possess today. We use a natural experiment, the colonization of Southeast Asia and Andaman Islands, to estimate the rate at which phonemic diversity increases through time. Using this rate, we estimate that present-day languages date back to the Middle Stone Age in Africa. Our analysis is consistent with the archaeological evidence suggesting that complex human behavior evolved during the Middle Stone Age in Africa, and does not support the view that language is a recent adaptation that has sparked the dispersal of humans out of Africa. While some of our assumptions require testing and our results rely at present on a single case-study, our analysis constitutes the first estimate of when language evolved that is directly based on linguistic data.

## Introduction

A capacity for language is a hallmark of our species [1], [2], yet we know little about the timing of its appearance. Language appears in the archaeological record only recently, with the advent of lexicographic writing around 5,400 years ago [3]. Therefore, investigators have addressed the origin of language by studying the evolutionary history of anatomical features [4]–[7] and genes [8]–[15] that are associated with speech production. This research suggests that other *Homo* species had the ability to produce speech sounds that overlap with the range of speech sounds of modern humans, and that species such as Neanderthals possessed genes that, in humans, play a role in language. But we do not know whether these archaic hominins actually produced speech, and if so, to which extent it was similar to our capacity for language. As of now, the anatomical and genetic data lack the resolution necessary to differentiate proto-language from modern human language. Until this resolution is improved, we need alternative lines of evidence in order to better understand the timing of language origin.

Here, we use phonemic diversity data to date the origin of language. Phonemic diversity denotes the number of perceptually distinct units of sound–consonants, vowels and tones–in a language. The worldwide pattern of phonemic diversity potentially contains the statistical signal of the expansion of modern humans on the planet [16]. As human populations left Africa, 60–70 kya, and expanded into the rest of the world [1], [17], they underwent a series of bottlenecks. This serial founder effect has led to a clinal loss of genetic [18]–[20], phenotypic [21]–[23] and phonemic diversity [16] that can be observed in present-day human populations. African languages today have some of the largest phonemic inventories in the world, while the smallest inventories are found in South America and Oceania, some of the last regions of the globe to be colonized. The loss of phonemes through serial founder effect is consistent with other lines of evidence that indicate that phonemic diversity is determined by cultural transmission forces, rather than cognitive or functional constraints. First, phonemic diversity varies considerably among languages, and several languages function with a restricted number of phonemes. Rotokas, a language of New Guinea, and Pirahã, spoken in South-America, both have 11 phonemes [24], [25], while !Xun, a language spoken in Southern Africa has 141 phonemes. Second, as predicted by theoretical models linking cultural transmission and demography [26]–[28], phonemic diversity correlates positively with speaker population size [16], [29]. And finally, phonemic diversity also correlates positively with the number of surrounding languages [16], suggesting that phonemes, like other cultural traits, can be borrowed. Phonemic diversity not only evolves culturally, but it also evolves slowly [16]. That the languages outside of Africa might have not recovered their original phonemic diversity, despite thousands of years of history in their respective continent, and despite all the historical, linguistic and social factors that lead to linguistic change [30]–[36], suggests that phonemic diversity changes over long time scales. Here, we take advantage of the fact that phonemic diversity evolves culturally and slowly, and use it as a slow-clock to date the origin of language.

By focusing on phonemes rather than cognates–words that share a common ancestry–we are able to circumvent problems that prevent current historical linguistic approaches from tackling the problem of dating the origin of language. Glottochronology uses the number of cognates that languages share to estimate when they diverged [37]–[39]. However, because cognates change over short time scales, the time-depth resolution of glottochronology is limited to a few thousand years [8]. Several historical, social and demographic factors influence cognate evolution, [31], [32], [34], [40], [41], a main one being frequency of word use. Common words evolve more slowly than rare ones [42]. Frequency of word-use alone predicts 50% of the variation in rates of cognate change, and can generate cognate half-lives that range from 750 years to more than 10,000 years [42]. Such variation in rates of cognate change is problematic for glottochronology, because glottochronology assumes a constant rate of cognate change [43], [44]. The assumption of a constant rate of change can be relaxed by applying phylogenetic methods to cognate datasets. These methods are powerful tools for estimating the date of divergence of language families [45]–[47]. Nonetheless, the temporal scope of this method is, at least in its current state, too limited to address questions about the origin of language. For instance, the average word half-life among Indo-European languages is about 2,530 years [42]. Here we circumvent the problem of variation in rates by averaging rates of phoneme accumulation over a large spatial and temporal scale.

Given that languages accumulate phonemes over long time scales, we ask how long African languages had to have been around in order to reach their current phonemic diversity. We start by building two related mathematical models that describe two ways by which phonemic diversity can rise through time. In the first model, phonemic inventory increases linearly with time, while in the second model phonemic inventory increases exponentially. Then, we parametrize the two models with empirical data. Finally, we use rewritten forms of the models to estimate the time span over which phonemes would have had to accumulate in Africa.

We do not attempt to capture all the factors that influence phonemic inventory size. The state of our knowledge does not allow us to formalize the specific mechanisms by which phonemic diversity increases and decreases. Therefore, our models are agnostic about the particular mechanisms of change in phonemic diversity, and capture only the net effect of these mechanisms on phonemic diversity. We summarize this net effect as a single number, a rate of phoneme accumulation through time. Note that phonemic changes that occur within a language and that do not lead to a net change in the size of the phonemic inventory are not relevant to our analysis. The crucial assumption underlying our models is that the net effect of the factors leading to phonemic gain is greater than the net effect of those leading to loss. When this assumption is met, all other things being equals, phonemic diversity increases through time.

The method used in this paper to date the origin of language is built upon various assumptions that require further testing. An assumption underlying the empirical parametrization of the model is that human populations have lost phonemes through a drift-loss process during their expansion across the world [16]. However, this hypothesis is not widely accepted among linguists. Problems with the drift-loss hypothesis are discussed in a collection of commentaries published in Linguistic Typology [48]–[60] and Science [61]–[63]. Overall, these commentaries highlight the fact that, while Atkinson’s hypothesis remains viable, alternative hypothesis to the worldwide pattern of phonemic diversity have yet to be satisfyingly rejected [64], [65]. As we describe our method and material below, we specify the other assumptions that we have made and that also need further investigation to be validated. Despite these caveats, our approach constitutes a novel solution to the difficult question of dating the origin of language.

## Analysis

We start by estimating the rate at which languages accumulate phonemes. Controlling for distance from Africa, the phonemic diversity of a language depends on the speaker population size, the geographic area over which the language is spoken, and local linguistic diversity [16]. This suggests that new phonemes are more likely to appear in large populations. It also suggests that phonemes can be borrowed through contact between groups and languages [16].

**Figure 1:**
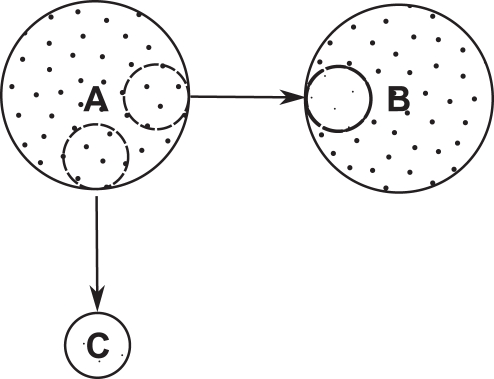
A model of change in phonemic diversity through drift and recovery. At time 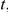 two small populations, *B* and *C*, emigrate from population 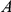 and colonize two different regions. Population *B* settles on a large landmass, and subsequently grows and diversifies linguistically. As a result, the average phonemic diversity of population *B* increases with time. Conversely, the phonemic diversity of population *C* remains stable through time because it occupies a small, isolated island. Therefore, the phonemic diversity of population *C* can be used to approximate what the phonemic diversity of population *B* would have been at time 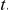 Large dots denote high phonemic diversity and small dots denote low phonemic diversity.

With that in mind, consider the hypothetical case of two small populations, *B* and *C*, that dispersed from the same parent population, *A*, *t* years ago (Figure 1). Suppose that *B* and *C* are similar in size so that they both experience approximately the same loss in phonemic diversity due to the founder effect. Now, suppose that population *B* colonizes a large continental territory and subsequently expands and diversifies linguistically [66], [67]. In contrast, population *C* settles on a small island that does not allow for population expansion and language diversification. Because of the differences between the regions colonized by *B* and *C*, population *B* will accumulate phonemes at a faster rate than population *C*. Furthermore, if population *C* evolves on a sufficiently small island and remains isolated for most of its history, then the rate of phoneme accumulation in *C* will be low, and its phonemic diversity will remain approximately stable through time. Consequently, the present-day difference between the phonemic diversity of *B* and *C* can be attributed to the new phonemes accumulated within population *B*. Thus, the current phonemic diversity of population *C* has remained through time a good approximation of the original phonemic diversity of population *B*. When this is true, and if the date of colonization, *t*, is known, then it is possible to estimate the phoneme accumulation rate in a large population as
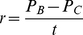
(1)assuming that phonemic inventories increase linearly, and
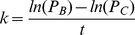
(2)assuming that phonemic inventories increase exponentially. 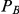 and 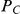 are the current phonemic diversity of populations B and C, and 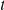 is the time elapsed between divergence of B and C, and the moment when their present phonemic inventories were measured. The linear model (Equation 1) is appropriate when phonemes increase independently of a language’s phonemic diversity. The exponential model (Equation 2) captures the alternative situation where the rate at which phonemes accumulate increases with a language’s phonemic diversity. Such dependence would arise, for instance, if each phoneme has the potential to give rise to new phonemes.

To estimate 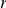 and 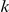 empirically, we take advantage of a natural experiment that approximates the scenario outlined in Figure 1, the migration history of humans in mainland Southeast Asia and the Andaman Islands. Both Southeast Asia and the Andaman Islands were colonized during the Pleistocene dispersal of modern humans out of Africa, a process that started 70–60 kya [71]. Genetic data indicate that humans dispersed in Asia following a coastal route, from India to Australia [17], [68]–[70], and that both Southeast Asia and Andaman Islands were colonized from a population that occupied the region spanning from southern India to the Malay Peninsula [69], [71], [72]. This dispersal was rapid. Genetic analyses estimate that it occurred approximately 65 kya [69], [71], and the archaeological record puts humans both in Southeast Asia [73] and Australia [74] at least 45 kya. Relative to the long temporal scale over which phonemes accumulate, we expect that the Andaman Islands and Mainland Southeast Asia were colonized simultaneously.

Populations in Southeast Asia and Andaman Islands differed demographically and linguistically. Like population *B* above, human groups expanded considerably after their arrival in Southeast Asia. By 40–20 kya, more than half of the total human population is estimated to have lived in South and Southeast Asia [75]. Today, about 160 million people live in Mainland Southeast Asia, and speak more than 60 languages. Conversely, we expect the Andaman population to have mirrored population *C* in the example above, and to have gained few novel phonemes, because of their low population size and remarkable degree of isolation. The Andaman Islands constitute a fragmented landscape of about 200 small islands, with a carrying capacity estimated to about 5000 individuals before contact with Europeans [76]. Genetic analyses suggest that the inhabitants of Andaman Islands have remained isolated since their arrival during the Pleistocene, up until the mid-19th century [70], [72], [77]. The 13 languages spoken on the islands at that time period are linguistic isolates, with no clear relationship to other Asian languages [78]–[81].

**Figure 2:**
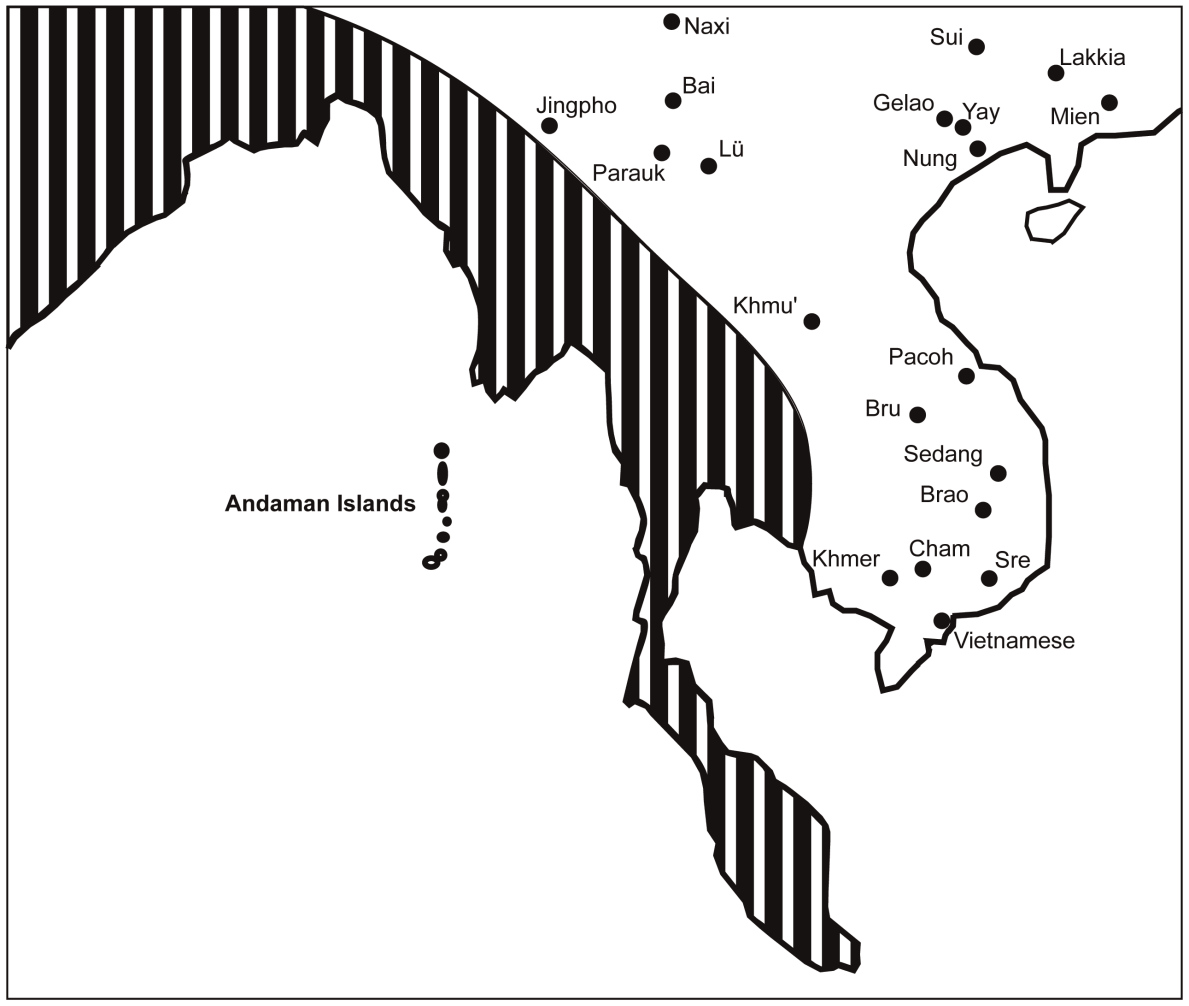
Approximate location of the languages included in the Mainland Southeast Asia sample. The languages located inside the shaded area were excluded from the sample because the region is a potential departure point for the colonization of Andaman Islands or Mainland Southeast Asia.

We estimate the parameters 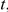 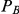 and 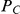 in Equations 1 and 2 as follows. Assuming that Mainland Southeast Asia and Andaman Islands were colonized at some point in time between 45 kya and 65 kya, we use 45 and 65 k as lower and upper bounds of 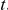 We obtained the phonemic diversity of languages of Mainland Southeast Asia and Andaman Islands using data from the UCLA Phonological Segment Inventory Database (UPSID) [24], [25]. While the categorical scaled measurements of phonemic diversity of the World Atlas of Language Structures (WALS) [82] were sufficient to detect a potential global serial founder effect [16], they are inadequate for the calculation of a phoneme accumulation rate. The UPSID contains the number of phonemic segments of a global sample of 451 languages. We estimate 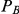 by taking the average phonemic inventory size of the languages in Mainland Southeast Asia. Assuming an eastward, coastal migration route, we have excluded the Asian languages that are located west of Andaman Islands (such as the languages from India and Nepal), as well as those spoken in Myanmar and the Malay Peninsula, because they could have served as departure points for the colonization of Andaman Islands (Figure 2). The 20 languages retained in our sample are thus those spoken in Cambodia, Vietnam, Laos and Southwest China (Table 1). The average phonemic diversity of the resulting sample is 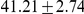 (errors represent one standard error). Great Andamanese (ISO 639-2: apq) is the only Andamanese language to appear in UPSID. Its phonemic diversity, 24, serves as our estimate of 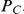


**Table 1:** Sample of Mainland Southeast Asian languages.

Language	ISO 639-2 code	Phonemic diversity
Bai	bca	29
Brao	brb	31
Bru Western	brv	42
Cham, Western	cja	32
Gelao	gio	43
Jingpho	kac	30
Khmer	khm	42
Khmu’	kjg	41
Lakkia	lbc	55
Lü	khb	31
Mien	ium	41
Naxi	nbf	49
Nung (in Vietnam)	nut	32
Pacoh	pac	33
Parauk	prk	77
Sedang	sed	55
Sre	kpm	37
Sui	swi	54
Vietnamese	vie	36
Yay	pcc	34
	Average	41.21
	Standard error	2.74

Setting 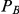 to 41.21 and 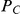 to 24, we obtain range estimates for the phoneme accumulation parameters 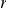 and 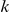 for a large, linguistically diverse population (Table 2). Note that, in the real world, we expect 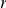 and 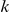 to vary through time and space, both within and between languages, as a result of various linguistic forces and historical contingencies. In contrast, our estimates of 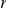 and 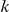 are averaged over 20 languages, that are dispersed over a vast spatial area, and that have been evolving in the region for perhaps as long as 60 ky. By using a time and space-averaged value, we are attempting to eliminate the effect of local contingencies and estimate the expected value of the rate of phoneme accumulation of human languages. We need such time and space-averaged value especially since we are dating an event that happened thousands of years ago, by using the average present-day phonemic diversity of multiple African languages.

**Table 2:** Phoneme accumulation rate estimates.

Time of colonization	Linear accumulation rate (*r*)	Exponential accumulation rate (*k*)
45 kya	0.38±0.06	(120.14±14.30)×10^−4^
65 kya	0.26±0.04	(83.17±9.90)×10^−4^

Estimates of the phoneme accumulation rate parameters for linear and exponential 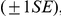 assuming that Mainland Southeast Asia and Andaman Islands were colonized 45 or 65 kya.

Using the rates of phoneme accumulation 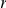 and 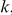 we calculate 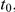 the time it would take for a language to acquire the phonemic diversity observed today in African languages, 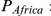


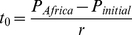
(3)or
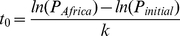
(4)where 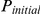 is the number of phonemes the first human languages started with. Phonemic diversity is assumed to have increased linearly in Equation 3, and exponentially in Equation 4.

To estimate 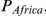 we use the average phonemic diversity of African languages that natively possess clicks [83], [84]. We do so because they comprise the African languages that have had the longest continuous history, and as a result are the ones that have lost phonemes due to founder effect the least recently. The largest language groups in Africa–Afro-Asiatic, Niger-Congo and Nilo-Saharan–underwent recently considerable geographic expansion [85], which could have decreased their phonemic diversity through serial founder effect. This idea is consistent with the fact that the average phonemic diversity of Afro-Asiatic, Niger-Congo and Nilo-Saharan languages is 36, 33, and 29 respectively, while the average phonemic diversity of African languages outside these families is 75. The African languages in UPSID outside of these three families are Hadza, Khoekhoe, Sandawe and !Xun. All of these languages use click consonants. Genetic analyses suggests that the speakers of these languages may have had the longest continuous population history [85]–[89], with mitochondrial DNA and Y chromosome variation indicating that the divergence between the click language speakers is at least as old as the divergence between any other pair of human populations [85], [86]. The main click language branches–Hadza, Sandawe and South African Khoisan (the last one includes Khoekhoe and !Xun) are estimated to have diverged as early as 55–35 kya [85], [86], with Hadza and Sandawe splitting 20–15 kya [85]. We have also included the Dahalo language in our sample. Dahalo is an Afro-Asiatic language, but the occurrence of click sounds in its core vocabulary suggests that it natively may have had clicks [90]. Using the five African click languages present in UPSID, we estimate 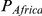 to be 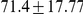 (Table 3).

**Table 3:** Sample of African languages.

Language	ISO 639-2 code	Phonemic diversity
Dahalo	dal	59
Hadza	hts	62
Khoekhoe	naq	41
Sandawe	sad	54
!Xun	knw	141
	Average	71.4
	Standard error	17.77

We cannot know what the initial number of phonemes of the first human language, 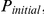 was. A reasonable assumption is that it is equal to the smallest phonemic inventory ever observed 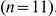 Therefore, we have set 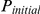 to 11 phonemes. On the other hand, it is possible that the languages with the lowest phonemic diversity today are outliers, and that a central value of the world’s phonemic diversity better approximates the initial phonemic diversity of human languages. We show how changing 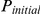 to the median phonemic diversity of the languages in the UPSID sample 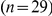 affects the result.

## Results

**Figure 3:**
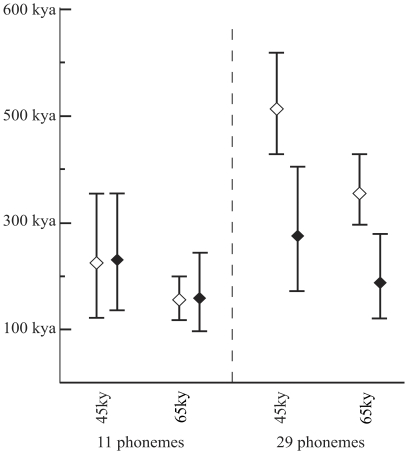
Date estimates for the origin of present-day languages. Range estimates are calculated assuming an initial phonemic diversity, 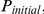 of 11 and 29, and a colonization time for Mainland Southeast Asia and Andaman Islands of 45 kya and 65 kya (parameter 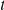). The lozenges denote central values, and the error bars represent calculations made with one standard deviation of the rate of phoneme accumulation (parameters 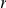 and 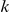), and the current phonemic diversity of African languages 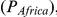 as described in the Results section. White and black lozenges represent results for the linear and exponential models of phonemic gain, respectively.

When 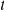 is 45–65 kya, the linear and the exponential growth models yield 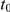 values of 232–159 kya and 225–156 kya, respectively. Setting 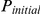 to the median phonemic diversity, 29, decreases our estimate to 163–112 kya and 75–108 kya for the linear and exponential growth models respectively. We have also estimated intervals around 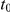 using one standard error around 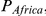 and the rates of accumulation 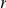 and 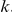 The value of 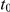 is minimized when phonemic diversity in Africa is low and phoneme accumulation rate is high. Conversely, 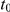 is maximized when phonemic diversity in Africa is high and phoneme accumulation rate is low. Therefore, the upper bound for 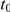 under linear growth is obtained by setting Equation 2 to 
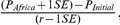
 and its lower bound is obtained by setting Equation 2 to 
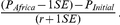
 Similarly, under exponential growth, the upper bound of 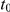 is 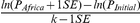 and the lower bound is 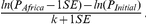 The resulting date ranges are shown in Figure 3.

These estimates are fairly insensitive to changes in model assumptions. We have considered the possibility that we are overestimating the phonemic diversity of African languages by restricting our sample to click languages. Click sounds may be evolving independently of non-click sounds. This would mean that a language could accumulate non-click phonemes at a certain rate, such as 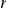 and 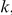 while simultaneously accumulating click-sounds at another rate 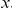 If this is true, then we cannot compare the African languages in our sample which have been accumulating clicks and non-click phonemes simultaneously, to Mainland Southeast Asian and Andaman languages which do not contain click sounds. To account for this possibility, we excluded click sounds from the phoneme inventory counts of African languages. The average non-click phonemic diversity of our sample of African click languages is 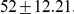 Using this value decreases our estimate to 158–108 kya and 187–129 kya for linear and exponential growth respectively. We have also tested the robustness of our results by excluding the Dahalo language from our sample of African languages. While Dahalo is thought to natively possess clicks, it is an Afro-Asiatic language [83] and as such might bias our sample of African languages towards lower phonemic diversity. Removing it from the sample increases our estimate to 244–167 kya and 230–159 kya for linear and exponential growth respectively. Finally, we also have increased our sample of Mainland Southeast Asian languages. Previously, we had excluded the languages spoken in Thailand, Malaysia, and Myanmar, as the colonizers of the Andaman Islands could have departed from one of these regions. By relaxing this assumption and including in our sample all the Mainland Southeast Asian languages contained in UPSID (the languages spoken in Myanmar, Thailand, Malaysia, Laos, Cambodia, Vietnam and Southwest China), we find that the average phonemic diversity in the region, 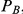 is 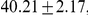 which increases our estimate to 242–168 kya and 236–163 kya for linear and exponential growth, respectively.

## Discussion

Our analysis suggests that language appears early in the history of our species. It does not support the idea that language is a recent adaptation that could have sparked the colonization of the globe by our species about 50 kya [1], [91]. Rather, our result is consistent with the archaeological evidence suggesting that human behavior became increasingly complex during the Middle Stone Age (MSA) in Africa, sometime between 350–150 kya [92]–[100]. However, we cannot rule out the possibility that other linguistic adaptations, that are independent of phonemic evolution, arose later and triggered the out-of-Africa expansion.

Our date estimate for the origin of language roughly coincides with the date range for the emergence of modern humans. Fossil evidence suggests that anatomically modern humans were present by 195–160 kya [101]–[104], and fossils classified as *Homo helmei*, that may be anatomically modern or nearly modern, are dated to 300–250 kya [95], [100]. Coalescence times from genetic data suggest that a genetic population bottleneck, possibly associated with a speciation event, occurred 200–100 kya [85], [105]–[108].

A population bottleneck causing a loss of phonemes would push back, or even reset the phonemic clock. As a result, our date estimates should be treated as minimum ages for the origin of language. It is thus possible that language arose before the last speciation event in our lineage, or even before the appearance of behavioral modernity.

Our date estimates should be treated with caution. Our results hinge on a series of assumptions in addition to the ones laid out in the Material and Method section. We assume that the rate of phoneme accumulation of Southeast Asia and Africa were similar. We assume that the Andaman languages did not accumulate new phonemes following the colonization of the Andaman Islands, or lose phonemes when their populations crashed upon contact with Europeans. We assume that the founding populations that settled Andaman Islands and Mainland Southeast Asia have lost an equivalent number of phonemes due to drift. Also, the UPSID phoneme counts do not include tonal distinctions. The absence of tonal distinctions in our data could add noise to our analysis, and bias it if it leads us to underestimate the phonemic diversity of one of the continental regions, Africa and Mainland Southeast Asia, more so than the other. We assume that the rate of accumulation of phonemes does not decrease as phonemic inventory size increases. An accumulation rate that decreases with phonemic diversity would lead us to underestimate the antiquity of present-day phonemic inventories. A similar bias would also occur if the phoneme accumulation rate changed through time as our species evolved. Furthermore, our estimate of the rate of phoneme accumulation is based on a single historical case. We are not aware of other colonization sequences that resembles the one outlined in Figure 1 that would also be ancient enough to allow for phonemic inventories to increase. However, despite the caveats we have highlighted here, this analysis constitutes the first appraisal of when language evolved to be based directly on linguistic data.
